# Surgery for Abdominal Wall Pain Caused by Cutaneous Nerve Entrapment in Children-A Single Institution Experience in the Last 5 Years

**DOI:** 10.5812/ircmj.8422

**Published:** 2013-02-05

**Authors:** Mirko Žganjer, Davor Bojić, Igor Bumči

**Affiliations:** 1 Department of Pediatric Surgery, Medical School Zagreb, Zagreb, Croatia

**Keywords:** Abdominal Pain, Propionibacterium Acnes, Child

## Abstract

**Background:**

Chronic abdominal pain (CAP) is a serious medical condition which needs to be approached with great attention. Chronic abdominal pain may be caused by entrapment of cutaneous branches of intercostal nerves (ACNES).

**Objectives:**

The aim of this study is the surgery for abdominal wall pain which caused by cutaneous nerve entrapment in children during last 5 years.

**Materials and Methods:**

In all children with ACNES, we tried conservative treatment with anesthetic and steroid injections. In children who were refractory to conservative treatment, we received surgical procedure like sectioning the entrapped nerve to obtain relief.

**Results:**

In 12 pediatric patients with chronic abdominal pain, we diagnosed ACNES. Each presented with abdominal pain and a positive Carnett sign. Local nerve blocks using anesthetic and steroid injections are the treatment. In all patients, we tried with local nerve block. In 3 patients, pain improvement occurs in the few minutes, and they were without pain after 5 days. In other 4 patients required a reinjection for pain recurrence. In one patients pain was gone. The maximum reinjection was 3. In other 5 patients, we did operative treatment like sectioning the entrapped nerve.

**Conclusions:**

Some children with CAP have ACNES. In all children with ACNES, we recommended local nerve blocks. If the local block in 3 times is not helping, neurectomy of the peripheral nerve is method of choice.

## 1. Background 

**Figure 1.**
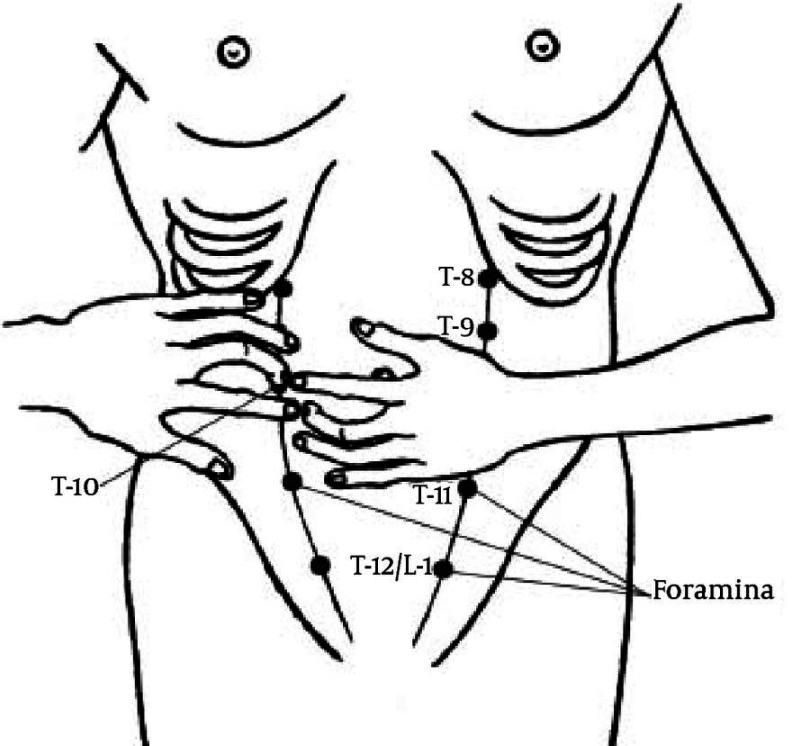
The Segmental Distribution of Skin Nerves in Front of the Abdominal Wall

Chronic abdominal pain (CAP) refers to the pain originating from the abdominal wall which is often misdiagnosed as arising from inside the abdominal cavity. When pain is chronic with no relationship to eating or bowel function but often relationship with posture, the abdominal wall should be suspected as the source of pain. Before the diagnosis was made, children were treated by pediatricians and made expensive and often unpleasant tests and treatment. When they did not find the cause of pain in the abdomen began to think about the pain at anterior abdominal wall. The first description of the above diseases was published in the international literature by JP Frank in 1792 (1). The first clear description of abdominal wall pain was provided in 1919. He thought that such pains could be mimicked by lesions that affected the vertebra, ribs, other associated muscles and they were the results of direct irritation of the nerves in the intercostal nerves by Cyriax (2). In many patients after gastroenterological diagnostic procedures diagnoses of patients referred as spastic colon, gastritis, depression, anxiety, irritable colon etc.(3). In most cases of chronic pain with no signs of gastroenterology diseases it is nerve entrapment at the lateral border of the rectus abdominal muscle (4-6). Carnett in 1926 recognized the diagnostic problems posed by abdominal wall lesions and maintained that pain could be caused by neuralgia affecting one or more intercostal nerves. Carnett's test is the key in a physical examination for diagnosing abdominal wall pain. Carnett’s sign is recognized in medicine as a method of determining the likelihood that the abdominal wall is the primary source of the pain and not the internal organs or viscera. The abdomen of the supine patient is palpated to elicit the area of tenderness. Then, with the palpating finger still located over the tender spot, the patient is asked to contract the abdominal muscles by raising the head from the examining table. Once the muscles are tensed, pressure is reapplied and the patient is asked if the pain has altered. If the cause of the symptoms is intra-abdominal, the tense muscles now protect the viscera and the tenderness should be diminished. On the other hand, if the source resides in the abdominal wall, the pain will be at least as severe or increased (7, 8). Kopell and Thompson noticed that peripheral nerve entrapment occurs at anatomic sites where the nerve changes direction to enter a fibrous or Osseo fibrous tunnel or where the nerve passes over a fibrous or muscular band and that entrapment can be at these sites because mechanically induced irritation is most likely to occur at these locations. Muscle contraction at these sites may add additional insult by direct compression, that traction on the nerve from muscle activity also is likely. Mechanical irritation causes localized swelling that may injure the nerve directly or compromise the nerve’s circulation. Tenderness of the main nerve trunk sometimes may be found proximal or distal to the affected portion (9). The thoracoabdominal nerves, which terminate as cutaneous nerves, are anchored in the rectus channel and in the skin. The most common cause of abdominal wall pain is nerve entrapment at the lateral border of the rectus muscle (10). The cause of pain is probably nerve ischemia caused by localized compression at the ring. Every reason for increasing pressure in abdominal cavity can directly compress the nerve and intensifies the pain. Pain trigger points frequently lie along the lateral margins of the rectus abdominal muscle (linear semilunar), where cutaneous nerve roots pass around the rectus sheath. The segmental distribution of skin nerves in front of the abdominal wall is as follows - T 6 and T 7 are located where ribs meet the edge of the rectus muscle; T 8 is at the junction of the rib margin and the lateral rectus; T10 is at the lateral edge of the rectus margin at the level of the umbilicus; T9 is halfway between T8 and T10; T11 is the halfway between T8 and T10; T12/L1 is at the level of the internal inguinal ring (8) ( Figure 1).

## 2. Objectives

The aim of the present study is the surgery for abdominal wall pain which caused by cutaneous nerve entrapment in children during last 5 years.

## 3. Materials and Methods 

**Figure 2.**
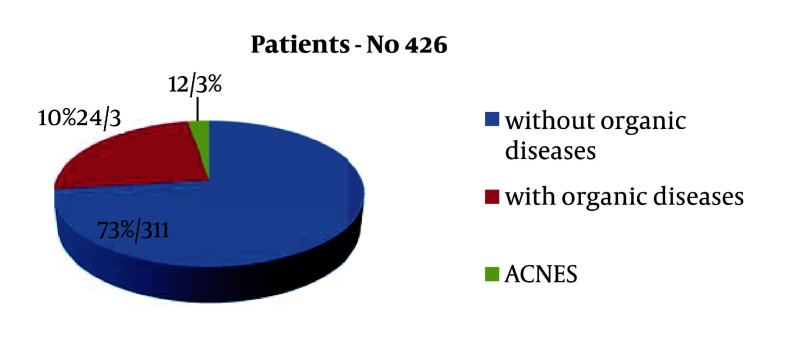
Patients reviewed the last 5 years with chronic abdominal pain in the abdomen

In the last 5 years, we had 12 patients with ACNES and who needed medical attention. In the last 5 years, we have had 426 patients who were treated for chronic pain in the abdomen. Of 426 patients, 311 had no organic disease; only 12 patients (2, 83%) had ACNES and should have additional treatment (Figure 2). Of the 12 patients, we had 5 girls and 7 boys. The median age was 15 years; the range of ages was between 11 and 17 years. In all children with chronic abdominal pain, we made Carnett test. In all children with ACNES, Carnett test was positive and in all other children with CAP, we did not find a positive Carnett test. All children with chronic abdominal pain are treated by gastroenterologists. All patients who are treated as ACNES, we asked whether they had an injury anterior abdominal wall. Only 5 patients were known to blow in the area of pain later. For more permanent relief of pain, it is often useful to inject a mixture of local anesthetic and corticosteroid.

Steroids presumably reduce inflammation or result in the thinning of connective tissue around painful nerve roots. In all 12 patients with ACNES, we attempted treatment with injections of analgesics (1mL of a 2% lidocaine solution). Lidocaine with steroids, we attempted in 3 patients where we believed that inflammation may be present. Frequent use of corticosteroids should be avoided because repeated injections or larger doses of the corticosteroid can cause thinning of the fascia and result in a hernia. None of the patients who had ACNES had surgery in the abdomen.

## 4. Results

Sometimes more than one injection may be required. In 3 patients, we had the disappearance of pain after the first injection, at 2 after the second and the 2 after the 3rd injection. In 5 patients, there were not improvements after 3 curative injections, and we had to do surgery (Table 1). It has been proposed that cutaneous nerve roots can become injured where they pass through the abdominal wall, perhaps by the stretching or compression of the nerve root along its course through the abdominal fascia. Then it is necessary to make the incision along the lateral margins of the rectus abdominal muscles (linear semilunar), where cutaneous nerve roots pass around the rectus sheath. Operative treatment consist sectioning and neurectomy of the entrapped nerve.

**Table 1.** Patients with Anterior Cutaneous Nerve Entrapment Syndrome and Treatments

	Values
**Gender**	
Girls	5
Boys	7
**Range of age (Mean)**	11-17 (15)
**Analgetics**	9
**Analgetics + steroids**	3
**Without pain after 1 injection**	3
**Without pain after 2 injection**	2
**Without pain after 3 injection**	2
**Operative treatment**	5

## 5. Discussion

Abdominal pain is overlooked problem in patients having chronic abdominal discomfort. These patients often go to the doctor and doing unnecessary tests, thus wasting time and spending money. Many authors have previously thought that the cause of chronic abdominal pain on anterior abdominal wall trauma or a consequence of surgery (9, 10). In our research, we realized that ACNES may be without previous traumatic event. Of all children who come three times a year in the hospital for pain in the abdomen, we recommended gastroenterological examination and treatment. In our hospital the past five years there were 426 patients who were treated by a gastroenterologist. 311 patients had no organic disease, 115 patients had a fault that required further monitoring and treatment. Of the 115 patients who had some symptoms, only 12 patients had ACNES. Acnes should be suspected when chronic pain is narrowing confined to a small area. After gastroenterological examination in most of cases, the diagnosis was made by patient history and physical examination, especially Carnett test. After the diagnosis, we move into the therapeutic process. All children are given an analgesic and it should start treatment. Injections of analgesics, with or without corticosteroids give at regular intervals of one month. For children whose pain does not stop after the third injection is indicated for surgery. Some physicians have recommended the use of corticosteroids as part of the local anesthetic injections (11). We are not supporters of corticosteroid therapy in routine use, because corticosteroids can cause severe pain in repeated applications, as well as atrophy of the tissues (12, 13). Lidocaine with steroids we attempted in 3 patients where we believed that inflammation may be present. The injection with analgesics serves two purposes, to relieve pain and reduce herniation of the neurovascular bundle through the fibrous ring. Occasionally, in absence of the relief of 3 injections surgical procedures like sectioning, the entrapped nerve obtains relief. Pain trigger points frequently seem to lie along the lateral margins of the rectus abdominal muscles (linear semilunar), where cutaneous nerve roots pass around the rectus sheath. It has been proposed that cutaneous nerve roots can become injured where they pass through the abdominal wall, perhaps by the stretching or compression of the nerve root along its course through the abdominal fascia. We've done a skin incision at the greatest pain. Pain trigger points frequently seem to lie along the lateral margins of the rectus abdominal muscles (linear semilunar), where cutaneous nerve roots pass around the rectus sheath. At the point of passage of the nerves around the rectus sheath, the nerves were cut in this point and solve the cause of pain (12, 13). We can still conclude that the chronic pain in the abdomen is a rare disease in which we think after we exclude other organic diseases. In patients with acnes, Carnett sign is positive. All patients should start with conservative treatment with analgesics and corticosteroids. If after three analgesic therapies no improvement is made, the surgical therapy and resection of compressed nerves roots are needed.
